# ASO Author Reflections: The Role of Postoperative Radiation Therapy Following Wide Excision of Neurotropic Melanoma of the Head and Neck: Now and into the Future

**DOI:** 10.1245/s10434-024-15657-3

**Published:** 2024-06-24

**Authors:** M. B. Pinkham, A. Herschtal, A. M. Hong, M. S.-T. Chua, R. A. Scolyer, S. Cumming, A. Pullar, J. Nobes, C. A. Barker, B. A. Guadagnolo, G. B. Fogarty, B. H. Burmeister, M. C. Foote

**Affiliations:** 1 Department of Radiation Oncology, Princess Alexandra Hospital, Brisbane, Australia; 2 University of Queensland, Brisbane, Australia; 3 School of Public Health and Preventive Medicine, Monash University, Melbourne, Australia; 4 Department of Radiation Oncology, Chris O’Brien Lifehouse, Sydney, Australia; 5 Melanoma Institute Australia, The University of Sydney, Sydney, Australia; 6 Genesiscare, Mater Hospital, North Sydney, Australia; 7 Department of Radiation Oncology, Peter MacCallum Cancer Centre, Melbourne, Australia; 8 The Sir Peter MacCallum Department of Oncology, The University of Melbourne, Melbourne, VC Australia; 9 Department of Tissue Pathology and Diagnostic Oncology, Royal Prince Alfred Hospital and NSW Health Pathology, Sydney, Australia; 10 Faculty of Medicine and Health, The University of Sydney, Sydney, Australia; 11 Charles Perkins Centre, The University of Sydney, Sydney, Australia; 12 Melanoma and Skin Cancer Research Centre, Monash University, Melbourne, Australia; 13 Queensland University of Technology, Brisbane, Australia; 14 Norfolk and Norwich University Hospital NHS Foundation Trust, Norwich, UK; 15 Department of Radiation Oncology, Memorial Sloan Kettering Cancer Center, New York, NY USA; 16 Department of Radiation Oncology, MD Anderson Cancer Centre, Houston, TX USA; 17 ICON Cancer Centre, Revesby, Australia; 18 GenesisCare Fraser Coast, Hervey Bay, Australia

Wide excision of neurotropic melanoma (NM) in the head and neck region is not always achievable and has been previously associated with an elevated risk of local recurrence. We performed a randomised trial to compare the effect on local relapse of adjuvant radiation therapy (RT) to the primary site to that of observation after excision.^1^ For inclusion, initial surgery required complete macroscopic removal of all visible disease with a ≥1 cm clinical margin (when practical) and ≥5 mm microscopically negative margin unless constrained by an anatomical boundary.

To our knowledge, this is the largest trial ever performed specifically in NM. However, it remained underpowered for the primary endpoint, because participant accrual was slower and the total number of local recurrence events in the control arm was much lower than anticipated. These results do not support routine use of adjuvant RT for NM. However, in cases where adjuvant RT might still be recommended (for example if ≥ 5-mm pathologic margins cannot be achieved or following re-excision of locally recurrent disease), this study provides useful data to show that generally long-term toxicity and quality of life is not negatively impacted.

Distant failure was the most common mode of recurrence, occurring in 18% (9/50) of participants. Adjuvant immunotherapy has now been shown to reduce the risk of recurrence and death following resection of high-risk stage II disease.^2,3^ The effect appears greater on distant rather than local or locoregional recurrence.^2,3^ NM with co-existing desmoplasia may also respond more favourably to immunotherapy than other melanoma subtypes and was an effective salvage strategy for localised recurrence in at least one study participant.^4^ SWOG 1512 reported a 56% pathological complete response rate following neoadjuvant immunotherapy for resectable desmoplastic melanoma.^5^ Future studies could investigate the benefit of adjuvant RT in patients with residual disease after neoadjuvant immunotherapy and surgery or compared with adjuvant immunotherapy alone in patients with resected disease and narrow or positive margins that cannot be improved.
